# Coach Training Within the Covid-19 Pandemic: Challenges and Potential Pathways

**DOI:** 10.3389/fpsyg.2021.570706

**Published:** 2021-04-29

**Authors:** Fernando Santos, António Cardoso, Paulo Pereira, Leisha Strachan

**Affiliations:** ^1^Escola Superior de Educação, Instituto Politécnico do Porto e Viana do Castelo, inED Centre for Research and Innovation in Education, Porto, Portugal; ^2^Escola Superior de Educação, Instituto Politécnico do Porto, Porto, Portugal; ^3^Faculty of Kinesiology and Recreation Management, University of Manitoba, Winnipeg, MB, Canada

**Keywords:** coaches, coach developers, sport, youth, development

## Abstract

In this article we aim to provide insights about the challenges stakeholders in Portugal and across the globe may face throughout the Coronavirus Disease 2019 pandemic to reorganize coach training programs and suggest strategies to help coaches learn. Such reflection may help stakeholders across socio-cultural contexts consider the consequences of the changes made to coach training programs, the rationale for these decisions and the need to acknowledge existent challenges such as lower course completion rates, pressures to deliver the curriculum and dilemmatic decisions about course format. Furthermore, we also suggest pathways for stakeholders to develop strategies that consider contextual variables and contribute to meaningful learning. Based on the Portuguese context, several issues are discussed.

## Introduction

The Coronavirus Disease 2019 (Covid-19) pandemic presents multiple social challenges such as confinement, social isolation, distance learning, and an increased dependence on technology (Basilaia and Kvavadze, 2020; Mishra et al., 2020). Specifically, universities and polytechnic institutes deal with many of the repercussions associated to the Covid-19 pandemic and have made multiple efforts to reorganize their curriculum and offer candidate youth sport coaches meaningful learning opportunities. However, these efforts come with several challenges, dilemmatic decisions, as well as both positive and negative consequences.

Considering the diverse ways coach training is delivered across the globe and the constraints posed by the Covid-19 pandemic, there is still the need to prepare youth sport coaches for the increasingly challenging task of helping youth learn values, life skills and sport-specific skills. Such mandate is not flexible and requires that all universities and polytechnic institutes thrive to set standards that may help candidate coaches become prepared fortheir future roles and responsibilities. Decisions concerning the delivery of coach education programs during the Covid-19 pandemic are challenging and have multiple complexities. Nonetheless, we question if these decisions should follow a market-driven logic whereas the key objective is to follow expectations created by candidate coaches and society in general with no consideration for ethics, values and negative consequences for learning processes. In the present article, we aim to provide insights about the challenges that stakeholders (i.e., administrators, university teachers, and coach developers) may face throughout the Covid-19 pandemic to reorganize coach training programs and suggest strategies to help coaches learn. Based on the Portuguese case, several issues are discussed. These reflections do not represent guidelines or recommendations for coach development systems, but instead reflexive questions that need to be posed moving forward.

## Pathways for Becoming a Coach

Across a wide range of countries universities and polytechnic institutes are responsible for developing coach training programs (Resende et al., 2016). For example, in the Portuguese context, there are diverse ways in which coach training programs are delivered. Some coach training programs are delivered by national and provincial sport organizations such as sport federations and associations. Furthermore, universities and polytechnic institutes may also certify coaches via graduate and post-graduate programs within the field of sport sciences. This is the prevalent approach across European countries, but also in other countries such as Brazil (Gaion et al., 2020).

Considering the impact of the Covid-19 pandemic there is the need to prompt a reflection about the changes made to coach training programs in the Portuguese context which, in some cases, have been detrimental for candidate coaches’ learning or extremely innovative and relevant. These decisions will have an important impact on (a) what coaches learn within the Covid-19 pandemic – they will be coaches and expected to coach in a context whereas youth have complex developmental needs; (b) their ability to foster sport-specific skills and skills throughout the Covid-19 pandemic and beyond; and (c) youth experiences and outcomes now and in the future (International Council for Coaching Excellence [ICCE] et al., 2014).

## The Current Context for Coach Development, Coaches and Athletes

Currently, it has become clear the Covid-19 pandemic will negatively impact youth’s developmental process on the short and long-term, specifically concerning their learning, mental health, and social life (Chaturvedi et al., 2021). Therefore, one of the main challenges for coaches is to help youth develop psychosocial skills such as emotional control and empathy that can be considered paramount for youth striving to develop within the Covid-19 pandemic. For example, nowadays youth deal with a vast array of situations such as e-learning classes and diverse tasks at home (e.g., helping parents take care of a younger sibling), as well as while managing other challenges created by the Covid-19 pandemic that are in constant mutation. It is important to note that Generation *Z* athletes have showed lack of social skills and resilience (Gould et al., 2019) which may be enhanced by the Covid-19 pandemic and by the lack of in-person interaction with coaches and peers, but also teachers and schools. Psychosocial skill development has been considered crucial for better mental health outcomes and prosocial behaviors (Graupensperger et al., 2021). Furthermore, athletes also have many other developmental needs that should be considered by coaches such as the lack of physical fitness (Dunton et al., 2020).

Therefore, there are several issues such as course formats, contents, strategies, and evaluation that should be addressed to better understand the impact of changes made to coach training programs and identify potential pathways moving forward for increased coach learning outcomes. Although the extent to which coaches, in many cases, may contribute to their athletes’ development is currently limited due to the confinement, coaches who take part in coach training programs during the Covid-19 pandemic still need to learn how to help athletes attain a range of outcomes. Such premise has guided coach development systems striving to rethink their approach toward learning and reorganize their curricula. Today’s reality and subsequently changes made to coach training programs across the globe are influenced by several pedagogical issues (i.e., what and how to teach coaches?), but also financial (i.e., what can be gained or lost through specific decisions?), and political ones (i.e., what aligns with public or private interest?) in the sense that pressures to avoid lower course completion rates and deliver the curriculum at all costs may exist. In the next section we discuss issues that stakeholders may need to bear in mind while making decisions about the structure and delivery of coach training programs.

## COVID-19 and Decision-Making in Coach Training Programs

### Asynchronous and Synchronous Course Formats

The urgency to continue to develop coach training programs via e-learning platforms and deliver coach certification programs should be paired with an urgency to make sure the learning opportunities provided serve coaches’ and athletes’ best interests and enable meaningful learning. With learning we do not mean exposure to information. Instead, we view learning in light of variables that lead to sustainable long-term coaching behaviors (Whitley, 2021). This an important distinction because stakeholders may have diverse understandings about what constitutes learning which, in turn, their decisions. Thus, a question needs to be posed: Is continuing to deliver courses enough for meaningful learning? We should consider that coaches educated within the Covid-19 pandemic will still be certified coaches and need to be able to contribute to their athletes’ development. One might even argue that today’s challenges are very pressing and complex. These candidate coaches require appropriate support from university teachers, coach developers and other stakeholders.

Therefore, it is relevant to discuss how courses have or may be delivered. For instance, coach training programs delivered partially or completely in an asynchronous manner (i.e., courses who include sessions that are not simultaneous or concurrent in time) should be carefully considered. In countries, such as Canada and United States of America, there is a culture of e-learning as multiple coach training programs and programs are already delivered through an online platform (Strachan et al., 2016). However, in countries such as Portugal where e-learning is not widespread and an uncommon tool for coach training programs, caution is needed. In other words, expecting candidate coaches to autonomously seek out materials, analyze them, master reflexive skills with no synchronous support and therefore learn may be unreasonable. The main challenge of asynchronous course formats is to provide solid grounds for learners to engage in meaningful tasks with no direct support from teachers. It should be noted that currently society in general, particularly candidate coaches, due to the importance of technology in our daily life, face the issue of widespread information. Further “… large-group lectures will almost certainly remain a common means by which higher education is delivered…” (Roberts, 2019, p. 73). Therefore, this context poses added challenges for candidate coaches who already struggle in being independent, autonomous, proactive, and who are not intrinsically motivated and open for learning. We should also consider that for those who face these challenges, asynchronous course formats may not contribute to meaningful learning. Conversely, synchronous course formats, although with direct support and instruction from facilitators, may also come with challenges such as engaging learners with the contents, limited communication, and active participation.

Thus, there may be value, in some cases, in combining asynchronous with synchronous sessions within coach training programs. Independently of the course format used, we should have in mind the course format alone does not automatically represent meaningful learning outcomes (i.e., a greater ability for coaches to foster specific outcomes and sustain coaching behaviors) and as mentioned previously just mean more information (Whitley, 2021). Nevertheless, we should consider that variables such as coaches’ background, previous experiences and age may also influence their ability to engage in e-learning and understand contents/materials through asynchronous and synchronous approaches (Paquette et al., 2019). Nevertheless, online teaching is a valuable avenue for meaningful learning when carefully and deliberately paired with appropriate learning strategies. The Covid-19 pandemic may serve as a stimulus for universities and polytechnic institutes to further reflect on how to effectively use online platforms to induce learning.

However, coach training, in many contexts, requires experiential learning opportunities and “learning by doing” (i.e., coaching and playing a given sport) which has been a crucial component for high quality training (Kolb and Kolb, 2005). In other words, online learning has limits and experiential learning could be a needed component. The balance between both online and experiential learning should be considered in a dynamic context and with candidate coaches’ needs in mind whereas predetermined answers and crystalized beliefs should not blear stakeholders’ judgments and decision-making. Additionally, pedagogy, funding and policy should interact with candidate coaches’ best interests (i.e., meaningful coach learning outcomes) in mind which is a challenge during the Covid-19 pandemic. However, pedagogical beliefs and convictions should not be limited and misconfigured due to financial interests or others.

The Covid-19 pandemic, in many cases, creates opportunities to have discussions around the best pedagogical approach and strategies utilized to help coaches learn how to foster athlete development which is positive. These components are key for effective coach training programs. As stated previously, simply exposing candidate coaches to materials probably does not represent enough support for meaningful learning, especially within the current situation whereas distance learning is the main source of learning. Therefore, we urge university teachers and coach developers to combine a range of informal and non-formal learning strategies such as in-class discussion forums, online communities of practice, as well as actively involve candidate coaches in reflections about real world problems and course material application (Lave and Wenger, 1991). For example, a coach might plan a session and infuse strategies to foster sport-specific skills and values. This session might be presented to other candidate coaches and prompt discussions about how to apply such strategies within competitive youth sport –such strategies need to be followed on a systematic basis. These reflections also have important implications for students who need to become available to move toward learner-centered approaches and embrace these demands.

### Assessment

Another issue that needs to be considered is how to assess coaches’ learning outcomes. Some online learning assessment tools, such as online tests, may not be valid to capture (a) coaches’ interpretations about course materials, (b) intentions for course material application, and (c) perceived behavior change. Thus, we need to pose two interconnected questions: What do we really want coaches to learn? And how do we want to assess learning? Although there are multiple challenges posed by the Covid-19 pandemic, past research has highlighted the need for coaches to learn how to set objectives based on youth’s developmental needs, as well as plan and implement activities toward these objectives (Camiré et al., 2018). This requires the ability to reflect on how to use knowledge and solve real world problems. It is a cause for concern if coach training programs neglect the need to teach and assess these components even throughout the Covid-19 pandemic. Again, we should reflect about the motivate for a different reasoning. Further, it is not feasible to replace meaningful coach learning with other understandings about coaching, learning and assessment that deviate from candidate coaches’ needs. If such deviation occurs effective programming may not be possible.

Assessment methods that evaluate coaches’ ability to become reflexive instead of leading coaches to reproduce contents, memorize concepts and frameworks may be needed (Lave and Wenger, 1991), specifically in nowadays changing and evolving coaching context. For example, coaches may be assessed by their ability to reflect on specific coaching situations or solve dilemmas within a community of practice. If coach training programs include traditional assessment methods during the Covid-19 pandemic such an approach might increase chances of educating coaches with no ability to apply course materials, as well as no autonomy and ownership for the training process and sustain coach behaviors on the long-term. The teaching model prelavent across socio-cultural contexts including in Portugal presents several issues, especially the lack of independence of candidate coaches and a coherent philosophy and pedagogical approach. There needs to be a common understanding between, but especially within universities and polytechnic institutes about assessment within coach training programs during the Covid-19 pandemic. Such discussion about assessment should reflect an understand about learning. We reiterate the need to answer the two questions raised in this section (i.e., What do we really want coaches to learn? and how do we want to assess learning?). Assessing coaches’ ability to reflect and envision potential pathways for course material application may be an important assessment piece. However, it should be noted this reflection piece derives from a specific understanding about learning and inherently assessment. Other universities and polytechnic institutes may have different understandings. Nonetheless, choosing assessment methods that are the most popular and/or easy to implement may diverge from candidate coaches’ best interests and are motive for concern. The logics of logistical, financial and political management need to be carefully considered within a pedagogy that highlights candidate coaches’ needs and where decisions lead to meaningful learning.

### Prioritizing Learning Instead of Delivering

Finally, one of the most important challenges faced by coach training programs nowadays and therefore by sport organizations, universities, and polytechnic institutes is to reorganize the curriculum, specifically what courses may be delivered or not (this is a possible outcome). Until this point, we have argued it is necessary to acknowledge that simply delivering coach training programs is not enough. We will proceed to explain why. There is an ethical and deontological obligation to make sure coaches receive appropriate training and support, and specific coach learning outcomes are attained. Coach training by itself is a complex endeavor that involves exposing coaches to a range of learning sources such as discussion forums, mentoring programs, and practicum opportunities (Paquette et al., 2019). Currently, in some cases, there might be the temptation to replace practical modules and coaches’ practicum with inequivalent tasks and requirements. Thus, we pose the following question: How can we replace practical modules and at what cost? We realize the trials and tribulations posed by the Covid-19 pandemic and the economic and social impact it has on certified and candidate coaches as well as universities/polytechnic institutes. However, we should also keep in mind that coaches will be able and considered prepared to coach youth across coaching contexts/age groups. Our role as researchers, university teachers and coach developers should be to develop novel and necessary changes to current coach training programs but keeping in mind that we cannot replace the irreplaceable and should be upkeep the highest rigor, values, and ethical conduct. For instance, replacing a 1-year practicum by a written report or project does not consider all evidence we have on the importance of experiential learning on coach learning. This reflection might need to be differentiated according to sport (e.g., individual, collective, outdoor, and indoor) due to the diverse ways the Covid-19 pandemic influences our day-to-day lives and, therefore, coach training possibilities. Despite this fact, the final questions for readers are: When are we losing sight of the true purpose of coach training? And what adaptations and changes are or not meaningful?

## Conclusion

In the present article, we aim to provide insights about the challenges that stakeholders (i.e., administrators, university teachers, and coach developers) may face throughout the Covid-19 pandemic to reorganize coach training programs and suggest strategies to help coaches learn. We acknowledge the need to develop changes to coach training programs in Portugal and across the world during the Covid-19 pandemic but suggest that decision-makers along with other stakeholders reflect on these changes and make informed decisions that consider existent knowledge on coach training and learning. A more thorough reflection on what can we do, what has been done and will be done is timely and necessary with regards to course format, pedagogical approach, and evaluation. In a time of crisis, coach training programs face many challenges that need to be addressed and can also help us reflect on how to change our understandings of learning and increase our effectiveness as teachers and coach developers.

## Author Contributions

FS, AC, and PP contributed to conception of the manuscript. FS analyzed the literature and wrote the first draft of the manuscript and took the lead on subsequent revisions. AC, PP, and LS wrote sections of the manuscript, revised subsequent versions, and provided conceptual guidance. All authors contributed to manuscript revision, read, and approved the submitted version.

## Conflict of Interest

The authors declare that the research was conducted in the absence of any commercial or financial relationships that could be construed as a potential conflict of interest.
